# Variation in resource competition traits among *Microcystis* strains is affected by their microbiomes

**DOI:** 10.1002/mlf2.12094

**Published:** 2023-12-18

**Authors:** Dylan Baker, Casey M. Godwin, Muhtamim Khanam, Ashley M. Burtner, Gregory J. Dick, Vincent J. Denef

**Affiliations:** ^1^ Department of Ecology and Evolutionary Biology University of Michigan Ann Arbor Michigan USA; ^2^ Cooperative Institute for Great Lakes Research, School for Environment and Sustainability University of Michigan Ann Arbor Michigan USA; ^3^ Department of Earth and Environmental Sciences University of Michigan Ann Arbor Michigan USA

**Keywords:** cultivation‐dependent, fitness, harmful algal blooms, host–microbe interactions, phytoplankton

## Abstract

Freshwater harmful algal blooms are often dominated by *Microcystis*, a phylogenetically cohesive group of cyanobacteria marked by extensive genetic and physiological diversity. We have previously shown that this genetic diversity and the presence of a microbiome of heterotrophic bacteria influences competitive interactions with eukaryotic phytoplankton. In this study, we sought to explain these observations by characterizing Monod equation parameters for resource usage (maximum growth rate *μ*_max_, half‐saturation value for growth *K*_s,_ and quota) as a function of N and P levels for four strains (NIES‐843, PCC 9701, PCC 7806 [WT], and PCC 7806 Δ*mcyB*) in presence and absence of a microbiome derived from *Microcystis* isolated from Lake Erie. Results indicated limited differences in maximum growth rates but more pronounced differences in half‐saturation values among *Microcystis* strains. The largest impact of the microbiome was reducing the minimal nitrogen concentration sustaining growth and reducing half saturation values, with variable results depending on the *Microcystis* strain. *Microcystis* strains also differed from each other in their N and P quotas and the extent to which microbiome presence affected them. Our data highlight the importance of the microbiome in altering *Microcystis*‐intrinsic traits, strain competitive hierarchies, and thus bloom dynamics. As quota, *μ*_max_, and *K*_*s*_ are commonly used in models for harmful algal blooms, our data suggest that model improvement may be possible by incorporating genotype dependencies of resource‐use parameters.

## Impact statement

Harmful algal blooms are increasingly affecting water quality and the economic and recreational value of freshwater and marine systems. A key factor determining whether such blooms can arise and persist is how well *Microcystis*, a cyanobacterium or blue‐green alga that predominates these blooms, competes with beneficial algal species. This study examines how both genotypic variation among *Microcystis* and bacteria associated with them can change characteristics of the host cell that determine the outcome of competition between *Microcystis* and other phytoplankton. Most notably, we showed how bacteria can improve the ability of *Microcystis* to grow at low nitrogen supply levels.

## INTRODUCTION

Harmful algal blooms (HABs) are a worldwide problem that threatens access to safe water for human consumption, recreation, and economic activities, as well as for wildlife^1^. Freshwater HABs are dominated by cyanobacteria that produce biomass in excess of what is consumed by zooplankton and thus disrupts the food web, and produce a diverse array of toxins^2^. These cyanobacterial harmful algal blooms (CHABs) are increasing in size and duration, fueled by the combined effects of land use change, invasive species, and increasing temperatures and an intensified water cycle due to climate change^3^, ^4^, ^5^. In light of a changing environment and to inform best mitigation policies^6^, it is important to have reliable predictive models of CHABs^7^. Current mechanistic models used to understand blooms and nutrients rely on parameterized relationships relating growth or yield to physicochemical parameters. Such models are typically informed by available data from laboratory experiments using isolates of cyanobacterial taxa, with an assumption that these parameters are representative of the dominant taxa in the community^8^, ^9^.

*Microcystis* is one of the predominant cyanobacterial genera in CHABs^10^. Genomic characterization of isolated *Microcystis* strains has shown that this genus encompasses extensive genotypic diversity, with most strains displaying >94% average nucleotide identity to other *Microcystis* strains. Based on commonly used species definitions, it could be considered to be a single species^11^, ^12^. However, species delineation within *Microcystis* has been suggested based on genomic analysis as well^11^, ^13^. Some of this diversity corresponds to the adaptation of *Microcystis* ecotypes to high‐ and low‐nutrient concentrations^14^, but most genotypic groups have yet to be associated with variation in abiotic or biotic environmental factors^12^. Similar to other bacterial genera and species with extensive within‐genus or ‐species genetic diversity, the observed genetic diversity among *Microcystis* strains should be able to explain variation in physiological traits measured among isolated *Microcystis* ^15^, ^16^, ^17^, ^18^. Variable traits range from growth responses to temperature and light conditions^19^, secondary metabolites produced^11^, resistance to predators^20^ and abiotic stressors such as reactive oxygen species^21^, and properties emerging from these traits such as their ability to compete with other phytoplankton species^22^, ^23^. This extensive genetic and physiological variation within *Microcystis* that can affect growth and death rates is not captured by biophysical and ecological models of bloom dynamics based on type culture parameters^12^. This may contribute to limitations of current models, for example, in their ability to predict bloom toxicity^24^.

It is worth explaining why explicit consideration of genetic and physiological diversity within *Microcystis* could be helpful to model improvement. We argue that this is in part because the ability to produce toxins, an important parameter models focus on, is not the main determinant of growth responses to environmental factors that are key for use in bloom severity and toxicity prediction. Hence, we need to better our understanding of how various trait values relate to each other, for example, the ability to produce toxins and the nutrient requirements or susceptibility to predation of a strain. Often, investment in one trait will come at the cost of another, and such trade‐offs are a common observation in ecology, for example, in investment in growth versus defense or requirements for nitrogen (N) and phosphorus (P)^25^, ^26^.

In addition to traits intrinsic to *Microcystis* based on its genome, differences in trait values observed between strains may derive from interactions between *Microcystis* and its microbiome, a consortium of associated microbes primarily consisting of heterotrophic bacteria^27^. In addition to heterotrophs, mixotrophic and autotrophic bacteria can reside in the *Microcystis* microbiome as well. For example, *Pseudanabaena* is commonly found to grow epiphytically on *Microcystis* colonies^28^. These communities are distinct from those residing in the surrounding water column or associated with other phytoplankton^12^, ^28^, ^29^, ^30^, ^31^, ^32^, they differ in function of *Microcystis* genotype^12^, ^14^, ^33^, and add functionality to *Microcystis* not encoded in its own genome^11^, ^14^, ^34^. In the context of our current study, microbiome impacts on competitive interactions between *Microcystis* species and other phytoplankton have been observed^22^. Whether the microbiome impacted competition with a green alga (*Chlorella sorokiniana*) depended on the *Microcystis* strain used. Microbiome impacts on host species interactions have been observed for plants and eukaryotic algae as well^22^, ^35^, ^36^, ^37^, ^38^. The mechanism of how microbiomes affect host interactions is not yet known.

Here we investigate the role of natural microbiota in influencing the nutrient uptake kinetics and storage by the cyanobacterium *Microcystis* in response to variable supplies of N and P. We focused on N and P because they are two key limiting nutrients in freshwater systems. We know that *Microcystis* strains vary extensively in cellular quota (atoms of N or P per cell), N and P uptake rates, and the response of growth rates to increasing N or P availability^12^, ^39^. We focus on the Monod equation parameters (maximum growth rate: *μ*_max_, the substrate concentration of the limiting nutrient at which half the maximum growth rate is achieved: *K*_*s*_) to characterize resource usage and the amount of each resource consumed by each cell *Q* ^40^ as they are commonly used in ecological and ecosystem models that simulate dynamics of different phytoplankton groups. We determined these parameters for N (as sodium nitrate) and P (as dibasic potassium phosphate) for four *Microcystis* strains belonging to different genotypic groups^12^. Considering the importance of competition for limiting nutrients and the ability of heterotrophic bacteria to remineralize organic nutrients to benefit host growth^41^, we included the effect of microbiomes on host nutrient requirement traits. We hypothesized that microbiomes alter *Microcystis* resource requirement traits in a host strain‐specific manner. This could help explain the *Microcystis* strain‐specific effects of microbiomes on competition with other phytoplankton that we have observed before^22^.

## RESULTS

### *Microcystis* strains differ in gene content underpinning traits relevant to this study

The *Microcystis* strains we used belong to the genotypic clusters Mae1 (PCC 7806 (wild type [WT] and Δ*mcyB*)), Mae3 (NIES843), and Mfl_ae (PCC 9701). As far as gene content is concerned, differences relevant to the present study are that: 1) only NIES‐843 and PCC 7806 (WT) carry the microcystin biosynthesis genes; 2) the number of P uptake proteins decreases from NIES‐843 to PCC 9701 to PCC 7806; 3) PCC 9701 and NIES‐843 have the *sbtA* gene as part of carbon (C) concentrating mechanism while PCC 7806 has *bicA*; and 4) PCC 9701 has the most extensive N uptake and metabolism gene repertoire (reviewed in Dick et al.^12^). While only point 2 and point 4 pertain directly to N and P uptake, we also report C quota (relevance of point 3), and microcystin is a N‐rich compound that may affect N uptake and storage (relevance of point 1).

### Microbiome composition analysis indicates genotype‐specific microbiome assembly

**Figure 1:**
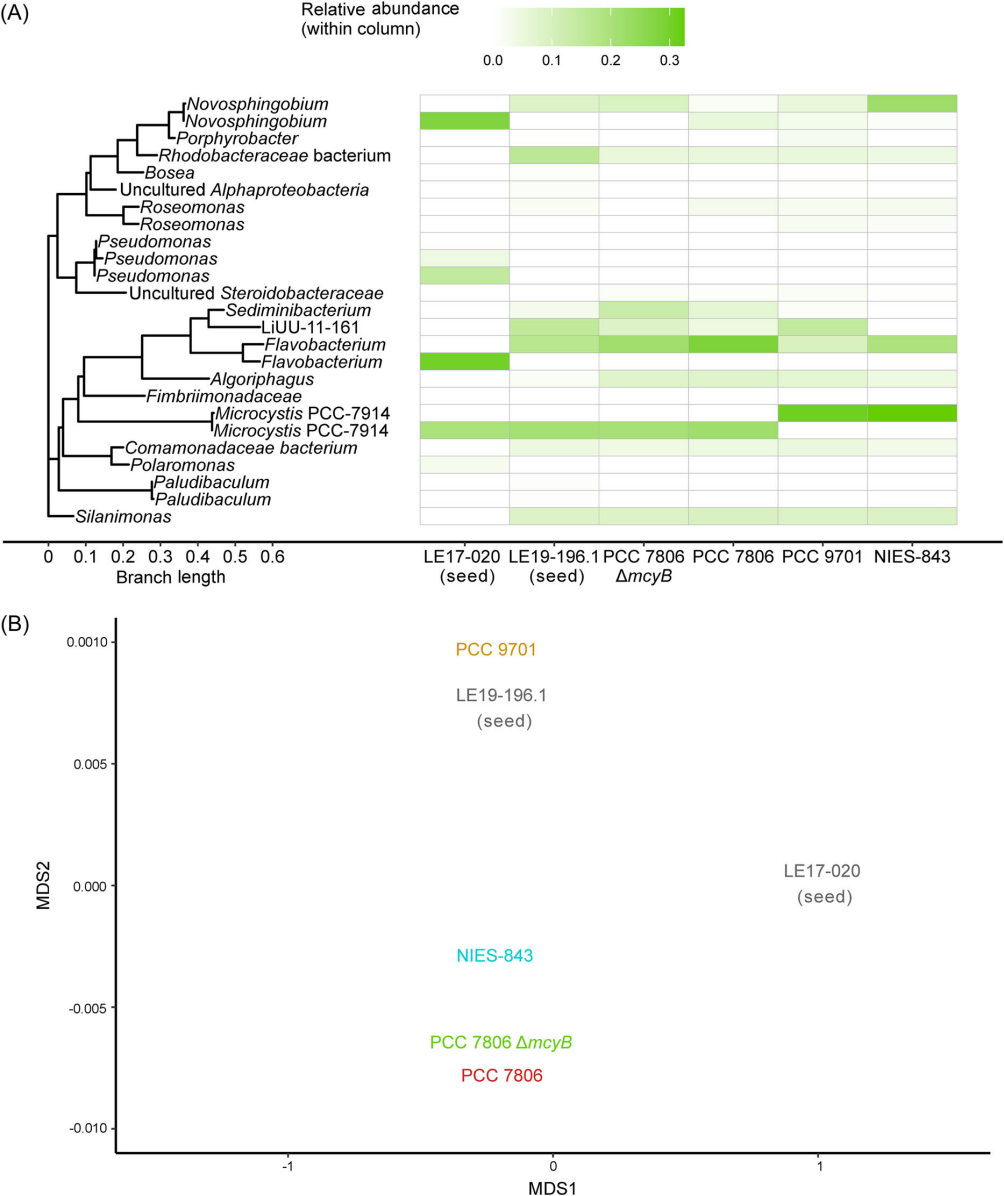
Phycosphere communities of created xenic and original seed *Microcystis* cultures. (A) Bacterial community composition, including the *Microcystis* host cells, determined by 16S rRNA gene sequencing of the V4 region. Heatmap colors indicate the relative abundance of ASVs ≥0.1% in at least one sample determined by DADA2. The seed cultures were filtered to 1.2 μm, combined, and added to the recipient axenic *Microcystis* strains, which each showed differences in the relative abundance of ASVs associated with them. (B) NMDS plot of phycosphere communities of the two seed *Microcystis* cultures (gray) and the four strains after exposure to the seed communities. The bacterial communities of mutant (Δ*mcyB*) and wild‐type PCC 7806 were nearly identical, whereas the bacterial communities of NIES‐843 and PCC 9701 were different from both the seed cultures and each other. Analysis was done in vegan (v2.6‐4) using a Bray–Curtis distance matrix. ASVs, amplicon sequence variant.

The two *Microcystis* cultures (LE17‐020 [Mae8] and LE19‐196.1 [unclassified genotype]) used as sources for the microbiome were dominated by a limited set of bacterial strains from the *Bacteroidota*, *Alphaproteobacteria*, and *Gammaproteobacteria* (Figure 1A). Each of the four *Microcystis* strains we used in our experiments, which were axenic, established a microbiome from the mixture of these seed communities that was unique in the relative abundance of these taxa. The wild type and mutant PCC 7806 strain microbiomes resembled each other the most (Figure 1A,B).

### Growth dynamics with respect to P limitation differ more between strains than between axenic and xenic versions of the same strain

**Figure 2:**
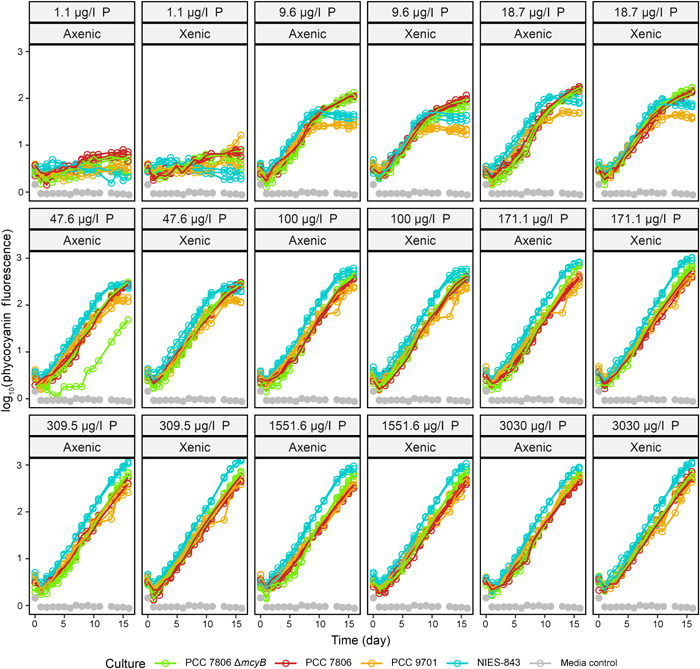
Growth curves of *Microcystis* strains based on phycocyanin fluorescence at varying P concentrations. In gray is the media control, while each color represents a different strain. Panels have been faceted by P concentration in PO_4_^3−^, and xenic or axenic culture status. Zero growth is seen at 1.1 μg/l PO_4_^3−^ except for xenic and axenic PCC 7806 and xenic PCC 7806 Δ*mcyB*. No further increase in growth rate was observed after 171.1–309.5 μg/l PO_4_^3−^ for all strains. P, PO_4_^3−^.

**Figure 3:**
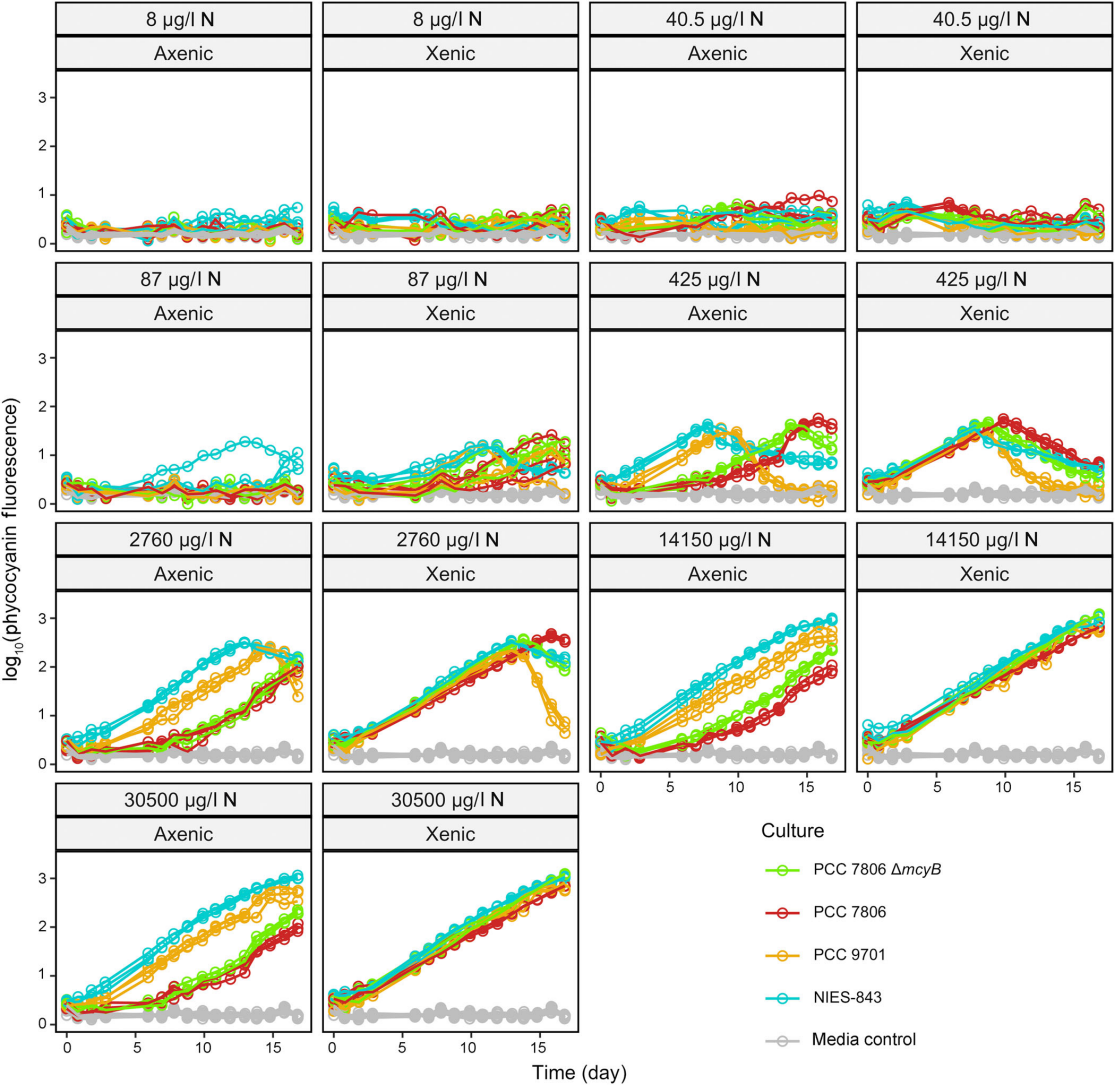
Growth curves of *Microcystis aeruginosa* strains based on phycocyanin fluorescence at varying N concentrations. In gray is the media control, while each color represents a different strain. Panels have been faceted by N concentration in NO_3_^−^, and xenic or axenic culture status. Zero growth was seen at 8, 40.5, and 87 μg/l NO_3_^−^ except for xenic strains at 87 μg/l NO_3_^−^, when growth started to become apparent. There was a noticeable lag in growth for the two axenic, but not xenic, PCC 7806 strains starting at 425 μg/l NO_3_^−^, but this was not observed in a trial experiment, suggesting this difference in lag phases was not a replicable result. Maximum growth rate was reached at around 2760 μg/l NO_3_^−^ for all strains. N, NO_3_^−^.

**Figure 4:**
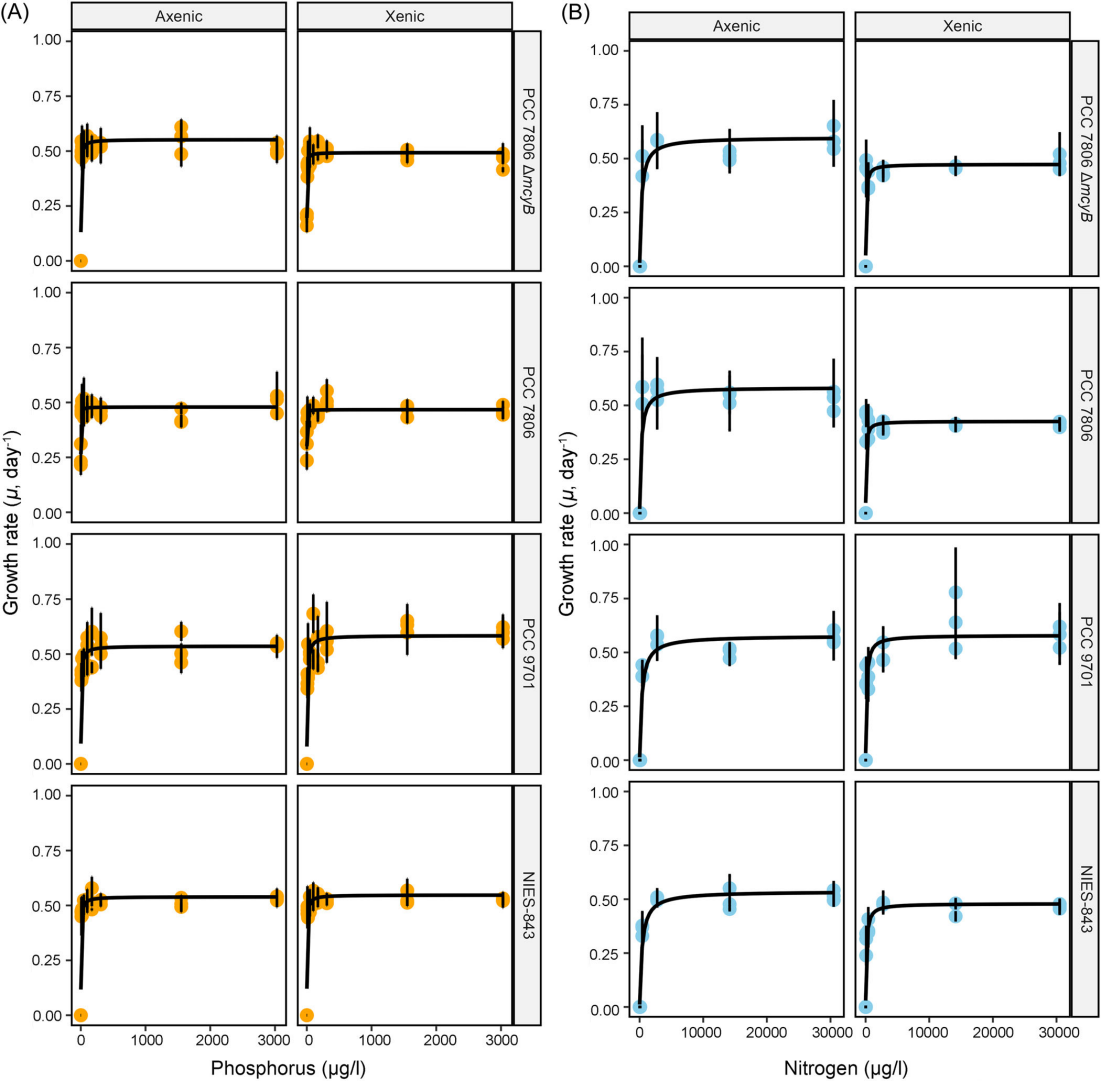
Response of each strain to increasing limiting nutrient concentrations and presence of a microbiome. Monod curves display the relationship between limiting nutrient concentration and maximum growth rate at that concentration for P (A) and N (B). Rows display the data for each of the four different *Microcystis* strains, while columns show the axenic and xenic versions of each strain. Points represent the average of the top five growth rates measured across measurement time intervals of the growth curve (±SE) for each replicate at each respective nutrient concentration.

**Figure 5:**
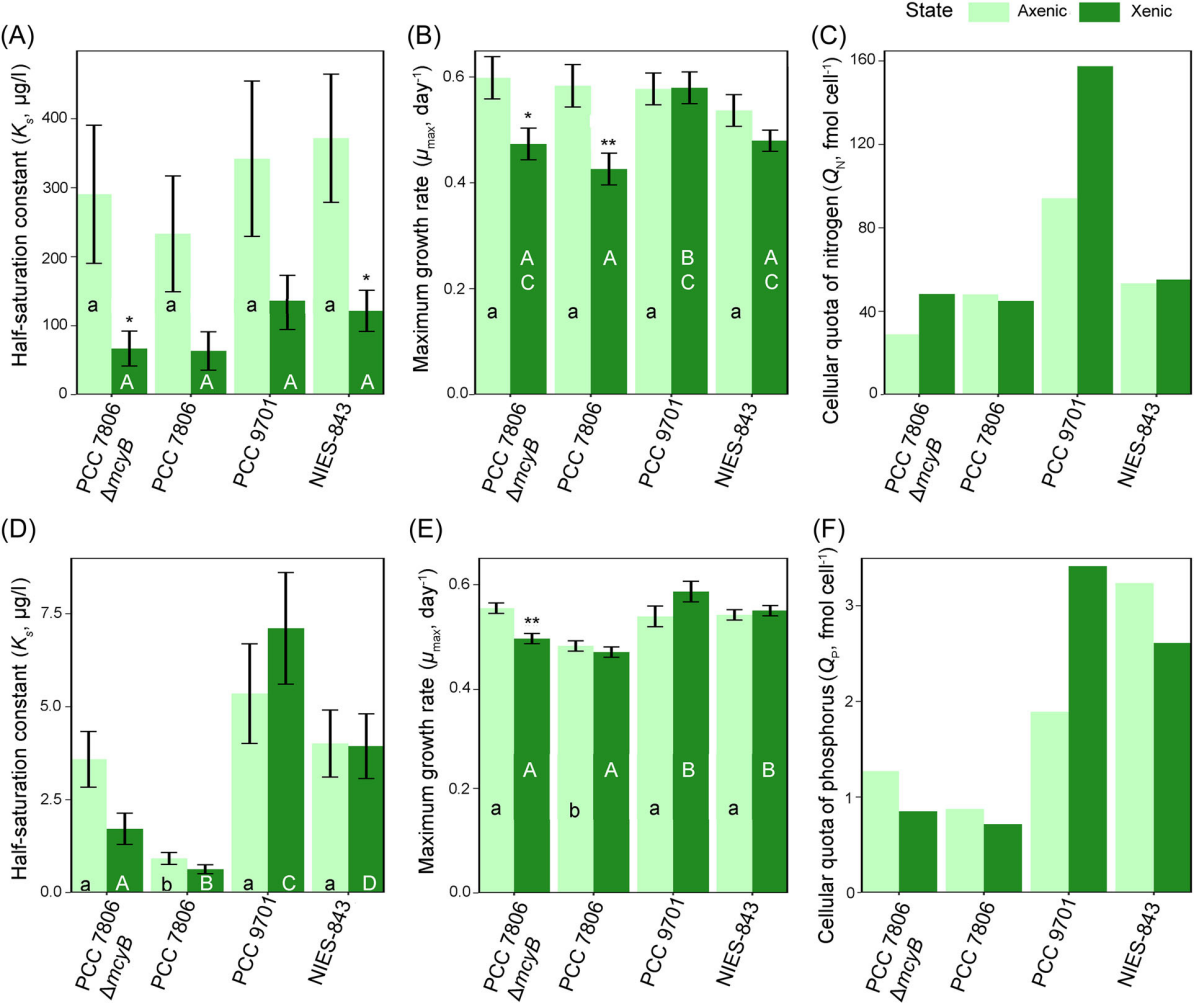
P and N growth parameters of *Microcystis* cultures in the presence and absence of a microbiome. (A) Half‐saturation constant (*K*_s_) of N (±SE). (B) Maximum growth rate (*μ*_max_) of N (±SE). (C) Cellular quotas of (*Q*_N_). (D) Half‐saturation constant (*K*_s_) of P (±SE). (E) Maximum growth rates (*μ*_max_) of P (±SE). (F) Cellular quotas of P (*Q*_P_) for four *M. aeruginosa* cultures in xenic and axenic state. *, ** Significant differences between *μ*_max_ and *K*_s_ values of the axenic (dark green) and xenic (light green) states of the same strain (*t*‐test with FDR correction; *p ≤ 0.05, **p ≤ 0.01, respectively). Lower‐ and upper‐case letters show significant differences between different axenic strains or xenic strains, respectively (*t*‐test with FDR correction, *p* ≤ 0.05). No significance was assessed for quota as only technical replicates were included.

The growth curves in function of varying levels of P limitation indicated no growth when no additional phosphate was added to the medium, except for PCC 7806 (WT, axenic and xenic, and mutant xenic), which showed limited growth at no P added (1.1 μg/l P total) (Figure 2; Figure 3 shows similar growth curves in function of N availability). Growth rates quickly increased with increasing P concentration, while maximum population size continued to increase until 171.1–309.5 μg/l. Differences in growth between strains were more noticeable at lower concentrations, particularly showing lower steady‐state fluorescence levels for NIES‐843 and PCC 9701. No clear differences between axenic and xenic versions of the same *Microcystis* strain were apparent. When fitting the Monod function to the fluorescence time series (Figure 4A), the intrinsic (i.e., values for the axenic strains) *K*_*s*_ values for P ranged between 0.92 and 5.35 μg/l P, while intrinsic *μ*_max_ ranged between 0.48 and 0.55 divisions per day, and the initial slopes ranged between 2.55 and 23.08 day^−1^ μM^−1^ (Table 1). Half of the between‐strain differences for *K*_*s*_ and *μ*_max_ were significant, based on a lower *K*_*s*_ and *μ*_max_ for the wild‐type PCC 7806 strain relative to all other strains, including the mutant (Figure 5D,E). We only observed a significant effect of the microbiome on the *μ*_max_ of the mutant PCC 7806 strain (Figure 5E and Table 1). However, the addition of a microbiome did increase the number of comparisons that were significantly different (Figure 5D,E), most noticeable that each strain had significantly different *K*_s_ from every other strain. The addition of a microbiome also increased the magnitude of between‐strain differences for both *K*_*s*_ and *μ*_max_ (Figure S2). We used these data to calculate R* values. R* is the substrate concentration at which growth rate equals death rate (we set the dilution rate at 0.2 day^−1^), and is a key parameter in resource competition theory to predict outcomes of competition, with the species with the lowest R* value being the best competitor for that resource. We found subtle changes in the R* values for P (P*; Table 1).

**Table 1:** Values (and standard error) of *μ*_max_ and *K*_*s*_ of the four *Microcystis* strains with (xenic) and without (axenic) a microbiome.

	P					N						Ratio		
Culture	*μ*_max_ (day^−1^) (SE)	*K*_s_ (μg/l) (SE)	Initial slope (day^−1^ · μM^−1^)	*Q*_P_ (fmol · cell^−^^1^) (SD)	P* (day^−1^)	*μ*_max_ (day^−1^) (SE)	*K*_s_ (μg/l) (SE)	Initial slope (day^−1^ · μM^−1^)	*Q*_N_ (fmol · cell^−1^) (SD)	N* (day^−1^)	*Q*_C_ (fmol · cell^−1^) (SD)	N:P	C:N	C:P
Axenic PCC 7806 Δ*mcyB*	0.55 (0.01)	3.58 (0.75)	4.77	1.27 (0.07)	2.0	0.60 (0.04)	290.57 (100.11)	0.029	28.86 (2.57)	145.3	176.14 (13.79)	22.72	6.10	138.70
Xenic PCC 7806 Δ*mcyB*	0.49 (0.01)**	1.72 (0.42)	8.91	0.85 (0.05)	1.2	0.47 (0.03)*	66.75 (25.29)*	0.099	48.22 (3.27)	49.4	263.6 (14.26)	56.71	5.47	310.03
Axenic PCC 7806	0.48 (0.01)	0.92 (0.16)	16.16	0.88 (0.38)	0.7	0.58 (0.04)	233.13 (83.95)	0.035	48.1 (2.87)	122.7	367.44 (23.97)	54.96	7.64	419.84
Xenic PCC 7806	0.47 (0.01)	0.63 (0.12)	23.08	0.72 (0.05)	0.5	0.43 (0.03)**	63.28 (27.84)	0.094	44.89 (2.33)	55.0	309.22 (16.14)	62.73	6.89	432.09
Axenic PCC 9701	0.54 (0.02)	5.35 (1.34)	3.11	1.89 (0.14)	3.1	0.58 (0.03)	342.09 (112.68)	0.024	94.16 (6.86)	180.0	587.91 (59.63)	49.81	6.24	311.01
Xenic PCC 9701	0.58 (0.02)	7.11 (1.50)	2.55	3.41 (0.31)	3.7	0.58 (0.03)	133.64 (39.25)	0.061	157.44 (11.43)	70.3	849.97 (62.65)	46.12	5.40	248.97
Axenic NIES‐843	0.54 (0.01)	4.01 (0.90)	4.17	3.24 (0.41)	2.4	0.54 (0.03)	371.77 (92.89)	0.020	53.33 (4.15)	218.7	304.92 (26.95)	16.47	5.72	94.18
Xenic NIES‐843	0.55 (0.01)	3.94 (0.87)	4.31	2.61 (0.27)	2.3	0.48 (0.02)	121.44 (29.81)*	0.055	55.12 (5.66)	86.7	305.29 (29.98)	21.11	5.54	116.92

*K*_s_, *μ*_max_, *Q*_P_, *Q*_N_, *Q*_C_, initial slope, and the calculated R* values or concentrations (based on *μ*_max_ and *K*_s_ values) at which net zero growth would be achieved in a chemostat setup with a dilution rate of 0.2 day^−1^ (P* and N*), and molar elemental ratios of C, N, and P for eight *Microcystis aeruginosa* cultures. *, ** Significant differences between *μ*_max_ and *K*_s_ values of the axenic and xenic states of the same strain (*t*‐test with FDR correction; *p ≤ 0.05, **p ≤ 0.01, respectively). No significance was assessed for quota as only technical replicates were included. *K*_s_, half‐saturation constant; *μ*_max_, maximum growth rate, *Q*_P_, cellular quota of P; *Q*_N_, cellular quota of N; *Q*_C_, cellular quota of C.

### The presence of a microbiome significantly changes the growth dynamics of *Microcystis* with respect to N limitation

Growth curves across varying nitrate concentrations indicated no growth at 8 and 40.5 μg/l N. We observed growth at the 87 μg/l N level for one axenic (NIES‐843) and all xenic strains, and we observed growth in all strains and conditions starting from the 425 μg/l N treatment (Figure 3). In most cases, both growth rates and yield increased until 2760 μg/l N. When fitting a Monod function (Figure 4B) to estimate the half‐saturation and maximum growth rate values, the intrinsic *K*_*s*_ values ranged between 233 and 371 μg/l N and *μ*_max_ between 0.54 and 0.60 divisions per day, with no significant differences between strains (Figure 5A,B). The initial slopes ranged between 0.020 and 0.099 day^−1^ μM^−1^ (Table 1). When comparing the growth of axenic and xenic versions of the same strain, the most noticeable differences were the lack of growth or lower growth rates of axenic strains at lower N levels, and the longer lag phase for axenic compared to xenic PCC 7806 strains (Figure 3). However, during pilot runs of the experiment, we did not observe this difference in the lag phase of the PCC 7806 strains (data not shown). In this context, it must be noted that the starvation step before the experiment, which ensures internal supplies of P or N were exhausted before growth in the defined nutrient concentrations, led to strains becoming chlorotic within 4 days in N starvation conditions, while not in P starvation conditions. The presence of a microbiome lowered the *K*_*s*_ by up to 250 μg/l N (significant change for 2/4 strains, Figure 5A) and *μ*_max_ estimates by up to 0.15 divisions per day (significant change for 2/4 strains) (Figure 5B). The impact of the microbiome on parameter values generally resulted in the between‐strain differences in *K*_s_ and *μ*_max_ to become smaller (Figure S2). When using these data to calculate R* values, we found that the presence of a microbiome decreased the intrinsic estimates for N* for a xenic strain to 30%–50% of the value for the corresponding axenic strain (Table 1).

### Nutrient quota varies by strain and is based on the presence of a microbiome

Measurements of cellular contents of C, P, and N indicated large differences between strains (Table 1). In addition, whether the microbiome affected quota was dependent on the *Microcystis* strain used. The P quota generally declined in the presence of the microbiome (Figure 5F), while the N quota generally increased (Figure 5C). C quota increased or remained similar, although the PCC7806 wild type and mutant did not display these patterns (Table 1). As only technical replicates were performed on a single culture, no statistical significance could be assessed.

### There was no evidence supporting an N/P trade‐off

To determine whether there are any apparent trade‐offs, we regressed *K*_*s*_, *μ*_max_, and quota values for N versus P. We observed positive, but nonsignificant linear correlations (Figure S3, *p* > 0.05).

## DISCUSSION

Within‐species trait variation has been shown to be an important factor in shaping community structure, food web interactions, and ecosystem process rates^42^, ^43^, ^44^. Within‐species trait variation is often linked to genetic divergence^45^, although phenotypic plasticity plays an important role too^46^. It is becoming increasingly clear that such phenotypic plasticity can be in part mediated by interactions with symbiotic partners^45^, ^47^, ^48^. In this study, we focused on the variation of key traits in resource competition within *Microcystis*. Understanding drivers of *Microcystis* nutrient‐use parameters is important due to its dominance in freshwater cyanobacterial blooms, an increasingly common issue worldwide due to cultural eutrophication and increased temperatures driven by climate change^4^. We assessed (a) differences between genotypes and (b) differences based on the presence or absence of an associated microbiome of heterotrophic bacteria. As observed before, we found strain variation for P and N requirements and cell quota traits between strains (reviewed in Dick et al.^12^). These previously observed differences were generally the result of both differences in *Microcystis* strains and activity of associated microbiome, though the effect of each was never explicitly considered. We showed that microbiomes can alter *Microcystis* maximum growth rates, and the half‐saturation values and cell quota measured for the *Microcystis*‐microbiome consortia, and do so more extensively for N than for P. The nature and magnitude of the effects were *Microcystis* strain‐specific, indicating that intrinsic competitive hierarchies between strains can be modified by their microbiomes.

### Differences between axenic *Microcystis* strains in resource‐use traits

The maximum growth rates we observed are on the higher end of reported values, which range between 0.1 and 0.6 divisions per day for our growth conditions at 20°C and 100 μmol photons m^−2^ s^−1^ ^12^. Variation for maximum growth rates among strains was limited compared to what has been observed among different *Microcystis* genotypes grown under the same conditions^14^. More variation was observed for the substrate concentrations at which half the maximum growth rate was obtained (*K*_s_), with much lower requirements for P than N and larger magnitudes of variation among strains for N (though more significant variation for P). We also noticed differences in yield based on fluorescence measurements, primarily at lower P concentrations, which aligned with differences in cellular quota (see next paragraph). Few values for *K*_s_ of *Microcystis* strains are available in the literature, in part due to the challenges with obtaining media with levels of P (and N) low enough to not sustain growth. We were successful at reaching such levels, and the initial slope of the Monod relationship for P and N was within the full range of previously reported data between 1.3 and 24 day^−1^ μM^−1^ for P^49^, ^50^ and in the low range of the previously reported data for N (0.014–1.3 day^−1^ μM^−1^)^51^, ^52^. Steeper slopes are typically indicative of a lower *K*_s_ value. The lower values for the calculated N* and P* values indicated that PCC 7806 was more competitive than PCC 9701 at slow growth rates, which is in line with PCC 7806 being more competitive than PCC 9701 with the green alga *Chlorella sorokiniana* in a previous study^22^.

Compared to the two key parameters determining competitive hierarchies discussed above, we showed more substantial variation between strains in the amount of N and P per cell (quota). All cultures were started from a standardized number of cells and allowed to grow for a similar amount of time, which considering the similarity in growth rates at the nutrient‐rich conditions provided, means that cultures were in a similar stage of growth upon sampling (late logarithmic phase). The higher cell quota for P in NIES‐843 and PCC 9701 corresponded with higher *K*_s_ values and lower fluorescence and presumably lower biomass yields at lower P concentrations compared to the two PCC 7806 strains. The higher P cell quota also was in line with the higher number of P uptake mechanisms identified in the genomes of the NIES‐843 and PCC 9701 strains. One possible explanation for these somewhat contradictory observations is that these strains have higher P demands, resulting in higher *K*s values despite higher cell quota and more uptake genes. The highest N quota was measured in the strain with the highest number of identified N uptake and metabolism genes (PCC 9701). As freshwater phytoplankton have been shown to display trade‐offs in their resource requirements for N and P, we used our data to determine if such a trade‐off was present in our data. Overall, comparisons of N and P data did not show a trade‐off between N and P requirements, although we made fewer strain comparisons than the larger data set in which this trade‐off was seen previously across phytoplankton species^25^, ^26^. Only a limited number of comparisons have previously been made between *Microcystis* strains grown in similar conditions, hence our data further emphasize the intrinsic within‐species variation in nutrient quota among *Microcystis* strains^53^, ^54^. Data on nutrient quota are important as (a) they determine how growth contributes to bloom biomass^55^, ^56^, and (b) they can inform how nutrient supply ratios affect toxin levels, which are generally N‐rich compounds^57^.

### Microbiome impacts on host plasticity in resource‐use traits

In addition to genetic variation, trait variation can also be caused by phenotypic plasticity in a single genotype. We focused on how the presence of a microbiome may induce phenotypic plasticity, which we rationalized based on the previous observation of how microbiomes can induce colony formation^58^ and affect competition^22^. Our data indicate that the microbiome can alter resource traits to an extent that they can change competitive outcomes as evidenced by large changes in R* for N due to the microbiome. We found that the effects of the microbiome were most pronounced for the minimal resource concentration at which growth occurred when N was limiting, which corresponded to lower *K*_s_ values, and we also found large shifts in the N and P quotas. The latter is in line with high plasticity in these traits previously reported in the function of growth conditions (reviewed in Dick et al.^12^), but our study adds to previous knowledge of the role the microbiome can play in altering cell elemental quota.

Effects on resource competition traits are not surprising as microbiomes are known to provide free nutrients from remineralizing organic matter or through N fixation^34^, ^59^, ^60^, ^61^. In fact, recent metatranscriptomic data from CHABs field samples support the idea that the microbiome indeed remineralizes organic N and makes it available to *Microcystis* ^62^. The effects of the microbiome on resource use traits can help explain why the presence of a microbiome mattered in competition experiments we carried out previously. We showed that the presence of a microbiome made *Microcystis* PCC 7806 and PCC 9701 more competitive against the green alga *Chlorella sorokiniana* ^22^. Ultimately, in the environment, *Microcystis* always has a microbiome associated with it. Yet, untangling the effects of intrinsic genetic differences between strains, and the extent to which extrinsic effects of the microbiome on trait variation augment or reduce these differences is helpful for improving our cyanobacterial harmful algal bloom models. Additionally, the composition of a microbiome has been shown to affect phytoplankton host physiological traits^63^, ^64^, ^65^, further emphasizing the need to understand intrinsic and extrinsic sources of trait variation. Whether microbiome composition matters for the resource competition, we assessed here remains to be determined.

The effect of the microbiome on resource traits was dependent on the host strain. This may be because of the intrinsic differences we identified between hosts in N, P, and C acquisition, and those inferred based on gene content differences for reactive oxygen defense traits. For the latter, it has been shown that, depending on the strain, associated heterotrophic bacteria can support the growth of *Microcystis* through ROS decomposition^21^. Additionally, it is possible that microbiome effects were *Microcystis* strain‐dependent if the composition of the microbiome we provided did not match each host's needs. Recent studies have highlighted the compositional diversity of *Microcystis* microbiomes and indicated complementarity between strain‐specific *Microcystis* auxotrophies and strain‐specific microbiomes^11^, ^12^, ^14^, ^34^, ^66^. Our data indicated that each *Microcystis* strain shifted the composition of the donor microbiomes differently, further supporting strain‐specific microbiome recruitment suggested before^12^, ^14^. However, as only a small number of heterotrophs were present in the donor microbiomes and considering the wide variability in *Microcystis* microbiomes reported thus far, the appropriate bacterial species to complement *Microcystis* needs may not have been present. Thus, different results may be possible with a different or more diverse donor pool. An intriguing question is that if the donor pool originated from a more nutrient‐limited lake, would bacteria affect *Microcystis* nutrient requirements differently? Our previous findings that the microbiome associated with *Microcystis* differed in function of lake nutrient status^14^ indicate that this may be a worthwhile area for future studies.

### Differences in observations between P and N assays

We observed smaller impacts of the microbiome on P parameters than N parameters. We expected impacts on P from the perspective that an element in lower absolute concentration would be more sensitive to changes in for example uptake kinetics. On the other hand, the fact that we observed larger impacts of the microbiome on Monod equation parameters for N may be explained by the much higher N requirements of cells. In the case of *Microcystis*, growth was only observed at levels of N at least eight times higher than P, and the cell quota for N was also 16–63 times higher, indicating high levels of plasticity in cellular stoichiometry, as previously reported (reviewed in Dick et al.^12^). Since N requirements are higher, it is logical that impacts on N acquisition were more noticeable. Another factor that helps explain the differences between microbiome impacts on growth under N versus P limitation is the fact that new inputs of N can be provided by associated N‐fixing bacteria, while this is not the case for P. We do not know whether any of the associated bacteria were N fixers, although some species of the abundantly present genus *Novosphingobium* have been found to do so^67^. In addition, bacterial N metabolism may have converted the supplied nitrate to the more preferred ammonium, which may explain enhanced *Microcystis* growth in presence of a microbiome. Future transcriptomic profiling of the *Microcystis*‐microbiome consortia relative to *Microcystis* alone and the microbiome alone could help us understand better which host and microbiome functions are underpinning our observations.

We also noticed that estimates for maximum growth rates tended to be slightly higher in the assays when N was the limiting nutrient compared to those for P, even though both were expected to be the same when both nutrients were plentiful. There is no biological reason why different essential resources must have the same theoretical maximum growth rates and this result is common among phytoplankton taxa^68^. The biological interpretation for this is that requirements for some resources saturate at a growth rate higher than the apparent maximum growth rate for the species under those conditions.

### Caveats and limitations

An important limitation of our study is that we used *Microcystis* strains that have been maintained in laboratory culture for an extended amount of time. Hence, they may not fully reflect the differences in nutrient responses that are present in the natural environment. One important trait of *Microcystis* in the environment, colony formation, is typically lost in long‐term culture and is indeed the case for the strains used in this study. Growth in densely packed colonies likely influences nutrient uptake dynamics and light acquisition, and the presence of tightly associated heterotrophic bacteria with these colonies has the potential to have important impacts on C, N, and P availability to *Microcystis* throughout the colony. Future studies using more recently isolated colonial *Microcystis* strains will help address this limitation and are currently underway using a set of isolates from Lake Erie^69^. Using newly isolated colonial *Microcystis* strains will also include strain‐specific associated heterotrophs that are likely more complementary to *Microcystis* strain‐specific metabolic needs, an issue we discussed above.

While our focus was on N and P use traits, it has to be noted that heterotrophic bacteria can also help overcome C limitation of a phytoplankton host^70^. While we cannot exclude this played a role in our observations, at lower nutrient concentrations, our data indicate that N and P were the limiting nutrients as intended, based on the clear and nutrient‐specific responses of growth rates to the concentration of N or P supplied.

Our quota calculation for the xenic state did not consider the cell numbers added by the microbiome as only *Microcystis* cells were counted. Hence, the xenic state quota likely represents an overestimate of the quota per *Microcystis* cell as part of the measured particulate nutrients was present in the associated bacteria.

Finally, we relied on the Monod model of growth in this study. While widely used, in part due to its simplicity, it has been shown that other models may represent growth in the function of resources better. In particular, the Droop model, which expresses growth as a function of internal storage rather than external supply, has been shown to model growth better, especially under fluctuating resource supplies^40^. Nonetheless, for the purposes of contrasting between strain differences in resource requirements and determining the impact of the microbiome on growth kinetics in function of available resources, our current approach sufficed as a proof of principle. Future studies would benefit from a transition to the Droop model.

In conclusion, we showed how associated microbiomes composed of heterotrophic bacteria can significantly modify intrinsic genetic differences in nutrient response traits that exist between *Microcystis* strains. Differences between strains and between xenic and axenic states were smaller for maximum growth rate than for half‐saturation values, and more noticeable for N than for P. Cell nutrient quota was affected by the presence of a microbiome as well. Storage of nutrients is a key factor affecting fitness and persistence in fluctuating nutrient environments, and points to an important area for further study as we continue to assess which features of within‐species variation we should consider for inclusion in bloom simulation and forecasting models. Taken together, the impacts of the microbiome were large enough to affect the intrinsic competitive hierarchies of the different strains, emphasizing the importance of the microbiome in determining the host's competitive abilities in the natural environment.

## MATERIALS AND METHODS

### Glassware cleaning and additional precautions

Initially, all glassware used for media stocks, fresh medium, pH adjustment, and *Microcystis* cultures were rinsed briefly with full‐strength sulfuric acid (H_2_SO_4_) in a fume hood, rinsed with Type I H_2_O, then combusted at 500°C in a muffle furnace to remove any contaminating residues. Glassware that had since been used was soaked overnight in a warm 2% Liquinox solution, then rinsed 6× with Type 1 H_2_O. Afterward, the glassware was placed in a 10% hydrochloric acid bath overnight, rinsed 6× with Type I H_2_O, filled with Type I H_2_O, and autoclaved on a wet cycle at 121°C for 30 min. Water was emptied aseptically in a biosafety cabinet before use. All cultures were grown in a modified COMBO medium^71^, minus the sodium metasilicate nonahydrate, due to high P contamination in this reagent. This did not impact *Microcystis* growth as silicate is not a required substrate for their survival^72^. Separate scoopulas were used when weighing out phosphate and nitrate compounds. COMBO medium was prepared in a biosafety cabinet, and pH was measured by taking aliquots and adjusting the original medium to a pH of 7.8. All media was filtered to 0.22 microns using a SteriTop PES filter (Millipore Sigma CAT # S2GPT05RE).

### N and P limitation assays

Critical to the determination of the Monod equation parameters is the removal of unintended sources of the limiting resource in the media. After optimizing glassware preparation and media component sources, we reduced the background levels of COMBO to 1.1 μg/l P (in the form of PO_4_^3−^), 8.0 μg/l N (in the form of NO_3_^−^), and 2.1 μg/l N (as NH_4_^+^).

Measurements of chlorophyll *a* and phycocyanin resulted in similar estimates of growth parameters (Figure S1), and considering signal strength for phycocyanin at low biomass levels was easier to distinguish from background levels than for chlorophyll *a*, we used phycocyanin data to track growth (Figure 2 for P limitation, Figure 3 for N limitation).

All axenic cultures were confirmed to be “bacteria‐free” beforehand with a 4′,6‐diamidino‐2‐phenylindole stain to identify any non‐*Microcystis* cells. Silicate‐free COMBO media was generated in a resource gradient for both N and P with the following concentrations: for N, concentrations were 8.0, 40.5, 87.0, 425.0, 2760.0, 14150.0, and 30500.0 μg/l N (in the form of NO_3_^−^); for P, concentrations were 1.1, 9.6, 18.7, 47.6, 100.0, 171.1, 309.5, 1551.6, and 3030.0 μg/l P (in the form of PO_4_^3−^). The concentration of P in the N‐limiting experiments was 1551.6 μg/l, and the concentration of N in the P‐limiting experiments was 14150.0 μg/l. Before the growth experiments, cultures were placed in P or N‐free media to deplete internally stored resources, which, in the case of N, led to the degradation of N‐rich photosynthetic pigments, which could be observed by the culture losing its green color. They were also washed to remove any nutrients released due to cell lysis. To accomplish this, *Microcystis* cultures in the late exponential phase of growth in silicate‐free COMBO medium were washed twice by spinning 5 ml of culture at 2200*g* for 10 min at 20°C and adding 5 ml silicate‐free COMBO containing zero nitrate or zero phosphate, depending on the resource being limited. The resuspended culture was put into an Erlenmeyer flask under these conditions: 30 µmol photons m^−2^ s^−1^, 16:8 h light:dark cycle, and 20°C and incubated for 3–5 days to allow depletion of intracellular N or P stocks. The wash step above was repeated again before inoculation onto the plate. Cell concentrations were standardized to 5000 cells per well (1 ml total volume) in triplicate and randomly distributed for each resource concentration in a Falcon 48‐well plate (CAT # 351178). No edge wells were used due to excess evaporation. The plates were placed into a light and temperature‐controlled incubator set to 20°C, on an orbital shaker at 60 rpm and illuminated with 100 μmol photons m^−2^ s^−1^, 16:8 h light:dark cycle. A beaker full of Type I H_2_O was used to humidify the chamber to help alleviate media evaporation. Plates were removed daily for chlorophyll‐a (ex 435, em 685) and phycocyanin (ex 630, em 660) fluorescence measurements using a Synergy H1 Hybrid Multi‐Mode Reader (Biotek Instruments, Inc.) and returned to the incubator in shuffled positions and locations to control for uneven lighting. Cell count aliquots were taken during exponential growth (estimated from fluorescence) and preserved at a 1% glutaraldehyde concentration in 0.2 ml tubes and stored at 4°C until counted.

### N and P quantification of COMBO medium

Concentrations of soluble reactive P (SRP), total dissolved nitrite and nitrate (NO_2_^−^ + NO_3_^−^), and ammonium (NH_4_^+^) were measured in duplicate via segmented flow analysis on a Seal AA3 AutoAnalyzer with AutoSampler (SEAL Analytical) according to the manufacturer's methods. SRP was measured using the molybdenum blue technique^73^. Briefly, ammonium molybdate and antimony potassium tartrate react with orthophosphate in an acid medium to form an antimony‐phosphomolybdate complex. The complex is then exposed to ascorbic acid, which produces a blue complex, and the absorbance is measured at 880 nm. NO_2_^−^ + NO_3_^−^ was measured by passing the water sample through a cadmium reduction column to reduce nitrate to nitrite, mixing with sulfanilamide followed by N‐(1‐naphthyl)ethylenediamine to form a red azo dye, the absorbance of which is read at 520 nm^74^. Ammonium was measured via a variant of the Berthelot reaction^75^, where dichloroisocyanuric acid, phenol, and sodium nitroprusside are mixed with NH_4_^+^ in the sample under basic conditions to form a blue iodophenol complex and the absorbance was measured at 630 nm. SRP and nitrate were standardized against NIST‐traceable standards (Hach Company) and ammonium was standardized against a certified standard solution (Hach Company).

### Creation of xenic microcystis strains

All cultures were kept in a growth room when not being used for experiments under these conditions: 30 µmol photons m^−2^ s^−1^, 16:8 h light:dark cycle, and 20°C, and refreshed every 2 weeks with 25 ml of fresh silicate‐free COMBO medium. Two nontoxic, unialgal Lake Erie *M. aeruginosa* cultures, LE17‐20 and LE19‐196.1 (described in detail in Yancey et al.^69^), were used as seed cultures to create xenic strains of four axenic *M. aeruginosa* strains: NIES‐843, PCC 9701, PCC 7806, and PCC7806 Δ*mcyB*. Overall, these source communities provided 23 non‐*Microcystis* amplicon sequence variants (ASVs), in line with community diversity observed in colony phycospheres in the environment, which mostly ranges between 10 and 40 OTUs;^28^ 15 ml of dense LE17‐20 and LE19‐196.1 cultures were vortexed for 10 s at maximum speed to loosen heterotrophs from *Microcystis* cells, filtered through a 1.2 μm RTTP filter to exclude any *Microcystis* cells, and combined into one tube and mixed thoroughly. The filtrate was divided equally among the four axenic *Microcystis* cultures above (in the late‐exponential growth phase) and supplemented with 10 ml of fresh silicate‐free COMBO. They were incubated in a light‐ and temperature‐controlled incubator set to 100 μmol photons m^−2^ s^−1^, 16:8 h light:dark cycle, at 20°C for 48 h. These cultures were used in N and P resource limitation experiments 5 and 7 months after the microbiome transplants, respectively, and were refreshed every 2 weeks with fresh silicate‐free COMBO medium during that time span^71^. Communities were captured on 0.22 μm PES filters for DNA extraction 8 months after the microbiome transplants occurred and snap‐frozen in liquid N_2_. In a study by Jackrel et al.^63^, algal microbiomes stabilized about 1 month after being seeded with bacterial populations from natural pond communities. Therefore, we expect similar microbiomes to have been present during both the N and P limitation experiments, 5 and 7 months postinoculation with Lake Erie *Microcystis‐*associated bacteria, respectively^63^.

### 16S rRNA gene sequencing and analysis

Filter DNA was extracted using the QIAshredder and DNEasy Blood & Tissue Kit (Qiagen), following the protocol for Gram‐positive bacteria. The V4 region of the 16s rRNA gene was amplified from each sample using a dual indexing sequencing strategy^76^. PCR products were visualized using an E‐Gel 96 with SYBR Safe DNA Gel Stain, 2% (Life Technologies cat# G7208‐02). Libraries were normalized using a SequalPrep Normalization Plate Kit (Life Technologies cat # A10510‐01) following the manufacturer's protocol for sequential elution. The concentration of the pooled samples was determined using a Kapa Biosystems Library Quantification kit for Illumina platforms (KapaBiosystems KK4824). The sizes of the amplicons in the library were determined using the Agilent Bioanalyzer High Sensitivity DNA analysis kit (cat# 5067‐4626). The final library consisted of equal molar amounts from each of the plates, normalized to the pooled plate at the lowest concentration. Library Preparation for Sequencing and Sequencing Libraries were prepared according to Illumina's protocol for Preparing Libraries for Sequencing on the MiSeq (part# 15039740 Rev. D) for 2 or 4 nM libraries. If the library concentration was below 1 nM, an alternative method was used for denaturation^77^. PhiX was spiked‐in at 15% concentration to 16S rRNA gene amplicon sequencing to create diversity. Sequencing reagents were prepared according to the Schloss SOP, custom read 1, read 2, and index primers were added to the reagent cartridge. Accuprime High Fidelity Taq (Life Technologies cat # 12346094) was used instead of the Accuprime Pfx supermix. Sequencing was performed by the University of Michigan Medical School Microbiome Core. Sequencing was done on the Illumina MiSeq platform, using a 2 × 250 MiSeq Reagent Kit V2 (Illumina cat# MS‐102‐2003), according to the manufacturer's instructions with modifications found in the Schloss SOP (https://github.com/SchlossLab/MiSeq_WetLab_SOP). FASTQ files were generated for paired end reads. Sequences were processed with DADA2^78^ and taxonomic annotations were determined with the SILVA release 138 QIIME2 classifier^79^, ^80^, ^81^ trained on the 515 F/806 R region. Details were recorded in an accompanying GitHub repository. A tree was built using FastTree (v2.1.10) using the GTR+CAT evolutionary model and run in sensitive mode (‐spr 4 ‐mlacc 2 –slownni options)^82^. Tree visualization was done with ggtree (v3.6.2)^83^ and NMDS plots were made in vegan (v2.6‐4) using a Bray–Curtis distance matrix of ASV abundances between samples (https://CRAN.R-project.org/package=vegan).

### Growth rate calculation, visualization, and statistical analyses

Analyses were conducted using the R Statistical language (version 4.2.2; R Core Team, 2022) on macOS Ventura 13.1 (http://www.rstudio.com/). Data import, manipulation, and plotting were done with the tidyverse (v1.3.2) family of packages (https://tidyverse.tidyverse.org/articles/paper.html)^84^. Growth rates were calculated for each time point as follows: the natural log of the quotient of the fluorescence intensity between consecutive time points was divided by the time difference between them. The five highest growth rates were averaged, and a standard error was calculated. To differentiate a true growth pattern resulting from increasing fluorescence versus spurious fluorescence spikes at individual time points, media control, and experimental fluorescence were log‐transformed and fit to a linear model. The media control slope and the experimental slope for each treatment were compared with a *t*‐test. A *p* < 0.05 showed that the experimental slope was significantly different from the control slope, indicating true increasing fluorescence. Conversely, a *p* > 0.05 signified that the experimental and control slopes were not significantly different, indicating zero growth in those treatments. This was a robust method of determining true growth, as only two out of 136 treatments were falsely flagged as nonsignificant. Statistical modeling for the *K*_s_ and *μ*_max_ was done using the functions nls and gnls in nlme (v3.1‐161) using the Monod equation $\mu =\mu_{\max}\cdot \frac{S}{S+K_{s}}$, with parameters *μ*_max_ and *K*_s_ allowed to vary by state and strain. *S* is the substrate concentration of a particular nutrient. The initial slope was calculated by dividing *μ*_max_ by the *K*_s_ (in μM). We estimated R* by setting the growth rate equal to a single dilution rate (0.2 d^−1^) in the Monod equation for growth, then solving for *S* ^85^. Nonlinear Regression with R, chapter 8, was used in this analysis^86^, ^87^. Contrasts of *K*_s_ and *μ*_max_ across strains were done using the function gnlht in aomisc (v0.650). All code and raw data are available in an accompanying github repository.

### Filtering for C/N/P quotas and total dissolved solids (TDS)

Four xenic cultures of *M. aeruginosa* were inoculated at a similar cell concentration in 235 ml of COMBO medium and placed in a light‐ and temperature‐controlled shaking incubator at 100 μmol photons m^−2^ s^−1^, 20°C, 60 rpm until phycocyanin fluorescence started to double within a 24 h period, which happened 8 days after inoculation. Aliquots were taken for cell counts on a hemocytometer. Then, a vacuum manifold at 5 psi was set up, and combusted Whatman GF/F filters were labeled and prepared for filtration, felt side up. For TDS, one replicate was filtered onto a 47 mm filter. For particulate P, two technical replicates were filtered onto 25 mm filters. For particulate organic C/N, three technical replicates were filtered onto 25 mm filters. Filter volumes varied based on culture density. All filters were rinsed 3× with Type I ultrapure water after initial filtering to remove C/N/P from the culture media, placed in a sterile petri dish, and kept at −20°C until processed. Beginning and end of run blanks were collected by filtering ultrapure water to control for any contaminating C/N/P from the filter. Particulate organic C and N filters were analyzed using a Carlo Erba NA 1500 Series combustion elemental analyzer. Particulate P filters underwent potassium persulfate and sulfuric acid digestion and then were colorimetrically analyzed using a SEAL Analytical AQ400 Discrete Analyzer.

## AUTHOR CONTRIBUTIONS

**Dylan Baker**: Formal analysis (lead); investigation (lead); methodology (equal); visualization (lead); writing—original draft (equal). **Casey M. Godwin**: Conceptualization (supporting); formal analysis (supporting); funding acquisition (supporting); methodology (supporting); supervision (supporting); writing—review and editing (equal). **Muhtamim Khanam**: Methodology (supporting). **Ashley M. Burtner**: Methodology (equal). **Gregory J. Dick**: Conceptualization (supporting); funding acquisition (equal); supervision (supporting); writing—review and editing (supporting). **Vincent J. Denef**: Conceptualization (lead); formal analysis (supporting); funding acquisition (equal); supervision (lead); writing—original draft (lead); writing—review and editing (lead).

## ETHICS STATEMENT

The authors declare they adhered to ethical standards for data generation and presentation and for authorship determination.

## CONFLICT OF INTERESTS

The authors declare no conflict of interests.

## Supporting information

Supporting information.

## Data Availability

Raw sequence data files were deposited to the NCBI SRA (BioProject PRJNA932314). Raw and processed data files, analysis, and visualization code is available on GitHub at https://github.com/mLife2023Baker.
