# Supplementary material for: Variation in resource competition traits among Microcystis strains is affected by their microbiomes
Source: mLife. 2023 Dec 18;2(4):401–15. doi: 10.1002/mlf2.12094 (PMC10989160; doi:10.1002/mlf2.12094)
Supplement: Supplementary file 1 — Supporting information. [file MLF2-2-401-s001.docx]

**Supplemental figures to Baker et al., submitted to mLife**

**
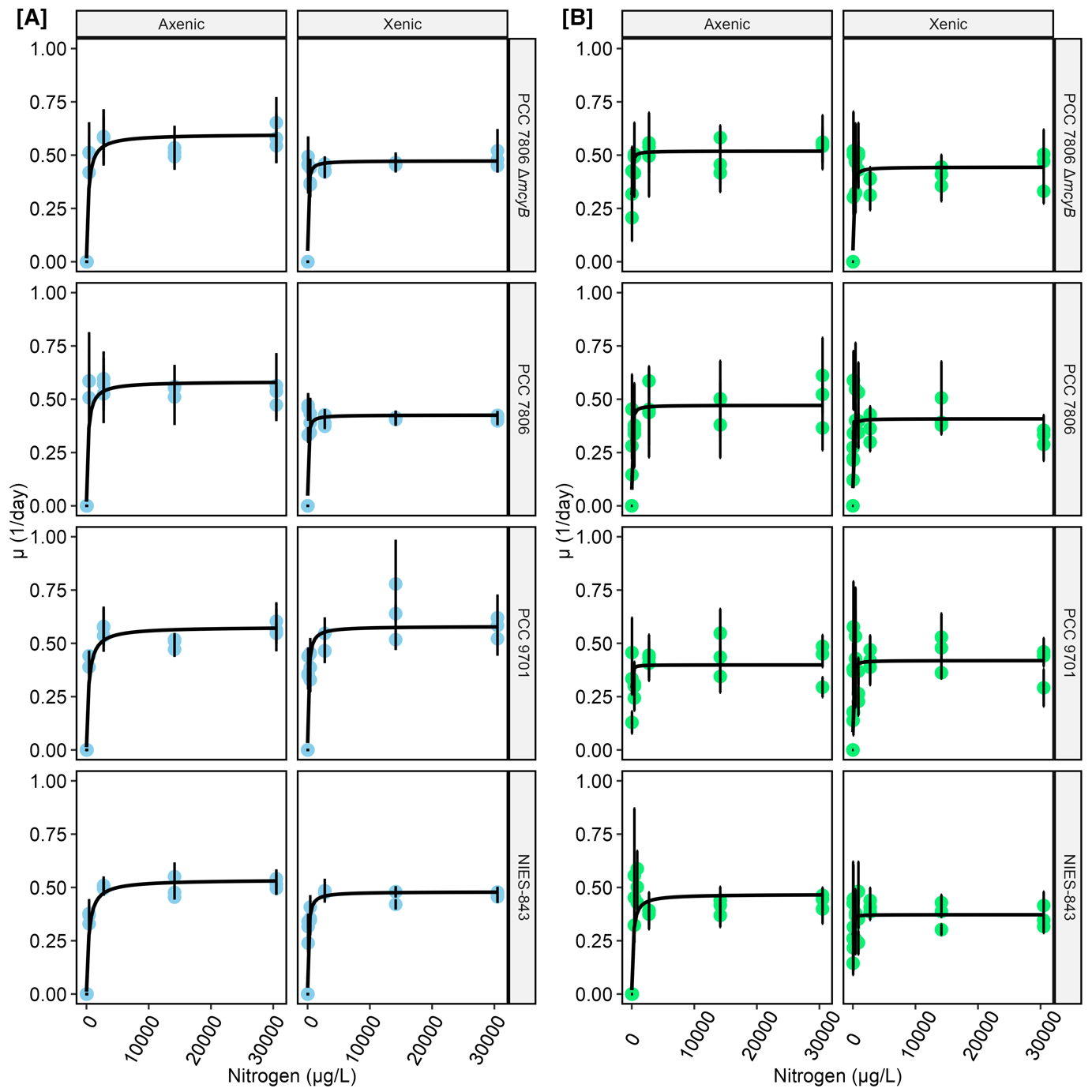
**

**Figure S1: Comparison of growth rates in function of substrate concentration derived from phycocyanin or chlorophyll *a* fluorescence.** (A) Growth rates derived from phycocyanin fluorescence measurements. (B) Growth rates derived from chlorophyll *a* fluorescence measurements. These data represent the average of the five highest growth rates for each replicate (± SE) under each growth condition. Phycocyanin fluorescence was more easily distinguishable from background than chlorophyll *a* fluorescence, especially at lower nutrient concentrations, and was more consistent between replicates. These two factors led us to use phycocyanin fluorescence for the final *K_s_* and μ_max_ parameter estimates.


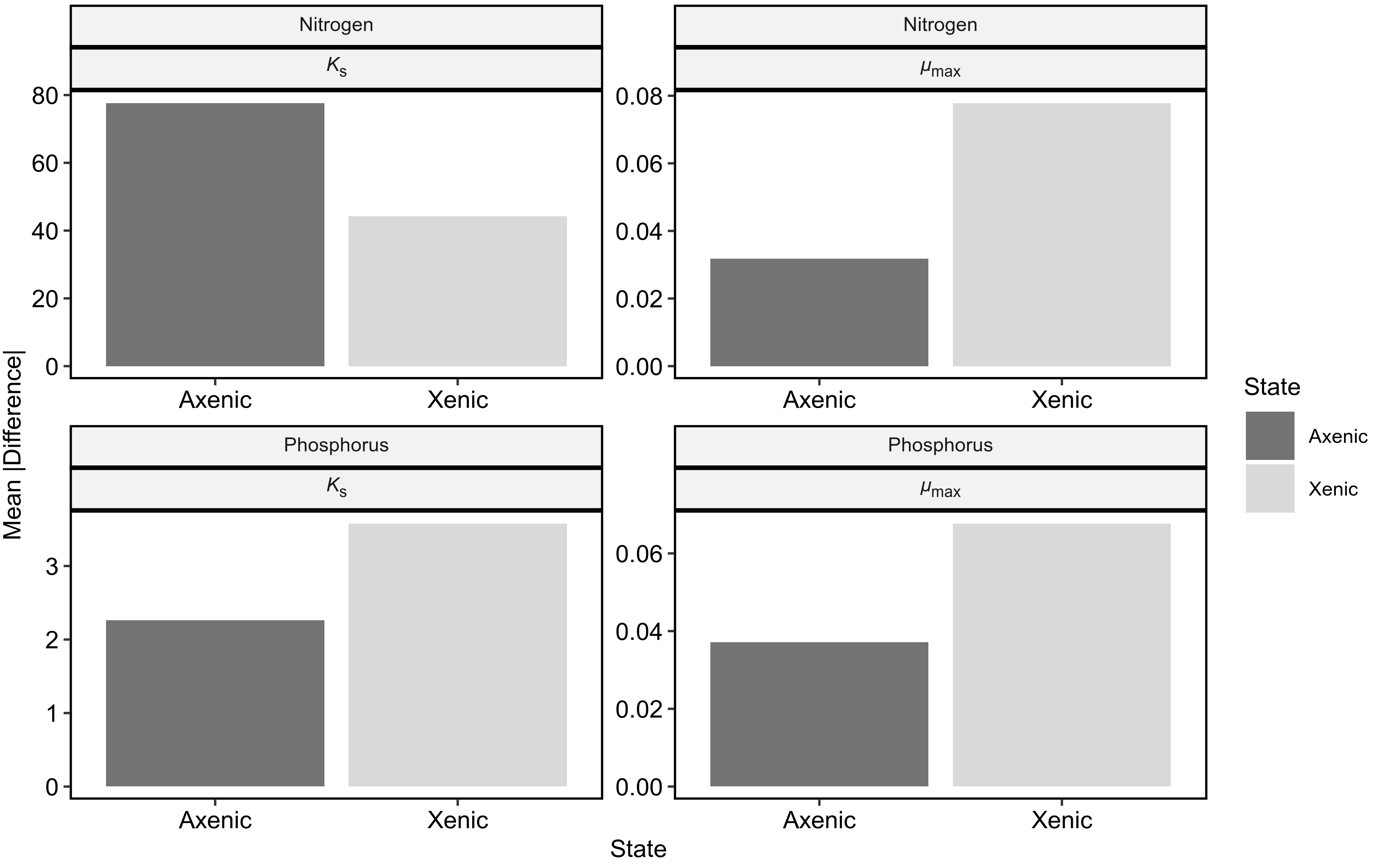


**Figure S2: Pairwise comparisons of μ_max_ and *K_s_* among the four *Microcystis* strains with (xenic) and without (axenic) a microbiome.** We calculated the average difference between Monod equation parameters of the 4 strains in axenic and xenic state, respectively.


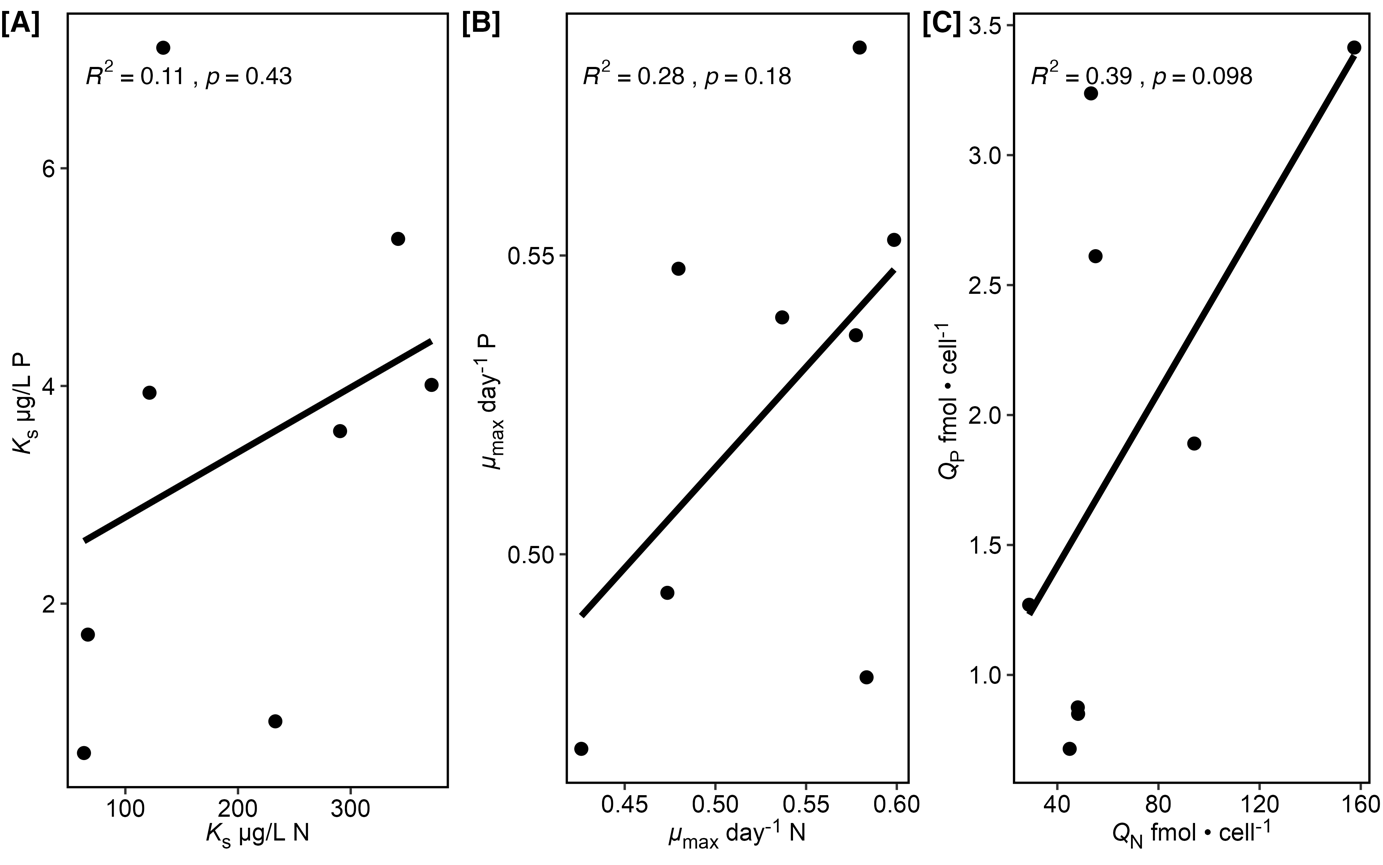


**Figure S3.** Regression analysis of nitrogen and phosphorus parameters (A) *K_s_*, (B) μ_max_, and (C) Quota measurements of the xenic and axenic *Microcystis* cultures. All correlations had a weak, non-significant positive correlation, p > 0.05.
